# Mind wandering at the fingertips: automatic parsing of subjective states based on response time variability

**DOI:** 10.3389/fpsyg.2013.00573

**Published:** 2013-09-05

**Authors:** Mikaël Bastian, Jérôme Sackur

**Affiliations:** ^1^Laboratoire de Sciences Cognitives et Psycholinguistique, École Normale Supérieure, CNRS, EHESSParis, France; ^2^Université Pierre et Marie CurieParis, France; ^3^Institut Universitaire de FranceParis, France

**Keywords:** mind wandering, subjective report, response times variability, Hidden Markov Models, time-course analysis

## Abstract

Research from the last decade has successfully used two kinds of thought reports in order to assess whether the mind is wandering: random thought-probes and spontaneous reports. However, none of these two methods allows any assessment of the subjective state of the participant between two reports. In this paper, we present a step by step elaboration and testing of a continuous index, based on response time variability within Sustained Attention to Response Tasks (*N* = 106, for a total of 10 conditions). We first show that increased response time variability predicts mind wandering. We then compute a continuous index of response time variability throughout full experiments and show that the temporal position of a probe relative to the nearest *local* peak of the continuous index is predictive of mind wandering. This suggests that our index carries information about the subjective state of the subject even when he or she is not probed, and opens the way for on-line tracking of mind wandering. Finally we proceed a step further and infer the internal attentional states on the basis of the variability of response times. To this end we use the Hidden Markov Model framework, which allows us to estimate the durations of on-task and off-task episodes.

## Introduction

Mind wandering refers to the occurrence of task-unrelated and stimulus-independent thoughts (Stawarczyk et al., 2011). In daily life, this spontaneous tendency of the mind to drift away from the here-and-now occurs about 30–50% of the time, with surprisingly few differences regarding the task at hand (Killingsworth and Gilbert, 2010).

The literature has successfully identified general factors that modulate the amount of mind wandering, be they context-dependent or more persistent. However, as Smallwood (2013) notes, the overall amount of mind wandering may depend on both the frequency and the duration of episodes. For example, mindfulness training might lead to *shorter* episodes of mind wandering through enhanced awareness of their occurrence (Schooler et al., 2011), whereas global time spent mind wandering might be reduced in a demanding task due to a reduction of the *frequency* of the episodes.

To our knowledge no extant methodology enables us to disentangle frequency from duration of mind wandering episodes. Up to now, mind wandering has been mainly accessed through discrete thought sampling: participants are randomly probed about their subjective states. This method only assesses mind wandering at the moment of the probe, but that tells us nothing about the time-course of the alternating states.

As an attempt to overcome this issue, participants could be asked to estimate the time spent mind wandering. Time estimation of conscious thoughts have already been reported (Klinger, 1978; Klinger and Cox, 1987), but lack secondary measures that would validate their reliability. This is critical, as one may be wary of any retrospective estimation of the time spent mind wandering on account of the dangers of complex introspection (Nisbett and Wilson, 1977; Johansson et al., 2006), and on account of the conclusive evidence that introspection is only faithful when retrospection—looking back to what has been done—and generalization—describing the mechanism instead of the occurrence—are kept to a minimum (Ericsson and Simon, 1980). Moreover, little is known about time estimation of mental events (but see Miller et al., 2010). Furthermore, among mental events, mind wandering is most often characterized by a lack of introspective awareness: participants often find out they have been mind wandering for some time without any previous acknowledgment of it (Schooler et al., 2011). It may thus be difficult for participants to estimate the duration of their mind wandering episodes when precisely they do not notice that they were mind wandering.

Similarly, we have no means of assessing participant's subjective states after a thought-probe. It has been suggested that spontaneous episodes of mind wandering might cease due to the interruption by a probe (Schooler et al., 2011), but there is in fact little evidence to this effect. It is even conceivable that the episode might start again right after the probe—to “terminate the thought.” In fact, reactive mind wandering (mind wandering about the fact that one has been caught mind wandering), has also been suggested (Cheyne et al., 2009). Hence, after a probe, participants could either continue their thought, restart their thought, have an other thought, or get back to focus. There is just no method that would help disentangling the different options.

Spontaneous “self-caught” reports of mind wandering may constitute an alternative to random thought sampling (Smallwood and Schooler, 2006). In this method, participant are requested to spontaneously report episodes of mind wandering as soon as they notice them. Unlike random thought probes, this method allows continuous tracking of mind wandering from the subject's perspective. However, this tracking crucially depends on awareness. Further, it can even be argued that monitoring one's own mind wandering is a task, and that as such, it is fallible, precisely because it is liable to mind wandering. Finally participants may set higher thresholds to spontaneously stop and report than to respond “yes, I was mind wandering” if probed (Bastian et al., submitted). Hence, the absence of spontaneous report of mind wandering is not sufficient to claim that the participant is not mind wandering: she might not judge her off-task experience salient enough, she might not be aware of it, or she might have forgotten to make the report. Therefore, even the self-catching procedure does not ensure a fully continuous assessment of the wandering mind.

To summarize, there is currently a deep methodological limit in the assessment of mind wandering: participants only tell us that they are mind wandering when we ask them to do so, or when they are themselves aware of doing so. Therefore, they report mind wandering at discrete time points that do not allow continuous tracking of their subjective state as they are experiencing it.

A crucial step to overcome this methodological issue may rely on the elaboration of a continuous index that would covertly track mind wandering. Behavioral [response time variability (Cheyne et al., 2009; Seli et al., 2012), increased error rate (McVay and Kane, 2012), decreased comprehension (Smallwood et al., 2008b)], electro-physiological [increased heart rate and galvanic skin response (Smallwood et al., 2007), pupil dilation (Smallwood et al., 2011)] and neural variables (increased activity in the default mode and executive networks (Christoff et al., 2009), increased energy in theta and delta bands and decreased energy in the alpha and beta bands (Braboszcz and Delorme, 2011), decreased amplitude of sensory-triggered ERP (Kam et al., 2011) have been suggested to be such indicators of mind wandering. However, crucially, all of these studies relied on contrasts between off-task and on-task periods, time-locked to discrete probes. Studies using random thought-probes (Christoff et al., 2009; Seli et al., 2012) opposed the few seconds preceding off-task reports to the few seconds preceding on-task reports. As for studies using spontaneous reports (Braboszcz and Delorme, 2011; Hasenkamp et al., 2012), they opposed seconds preceding and seconds following the spontaneous report, with the assumption that participants would be able to refocus immediately after the report.

While this approach seems a necessary step in the elaboration of an index of mind wandering, we suggest that a global analysis taking into account the full length of the experiment is now critical. But how can we extrapolate subjective states away from discrete moments when subjects report them? Here, we propose the following strategy: first, we design a candidate index of mind wandering: this index should both correlate with subjective states when these are available, and it should be based on objective measures that are available even when participants do not report on their subjective states. Next, we compute the index at every time-point in the experiment and identify regular patterns (namely peaks and troughs) in its time-course. We then test whether the temporal position of reports relative to these patterns is predictive of the content of the subjective report. We take the finding that temporal proximity to peaks of the index is predictive of mind wandering, *above and beyond its absolute value*, as an indication that the index carries information about the subjective state of the participant throughout the entire duration of the experiment.

In this paper, we applied this strategy to a re-analysis of data of three experiments (*N* = 106) based on the Sustained Attention to Response Task (Robertson et al., 1997). These data were not intended at first for this project and will be presented in full details in Bastian et al. (submitted).

So as to theoretically validate the analyses, we go one step further and propose a model of the fluctuations of mind wandering in our data. We conceptualize our participants experience during the experiment as a Markov chain of two attentional states: on-task and mind wandering. We show that, based on the assumption that variability of response times is heightened in the mind wandering state, we can parse the full time series of response times and reveal episodes of mind wandering. We show that this latent classification is both internally consistent and correlates with participants subjective reports.

## Methods

### Data and designs

The design of the three experiments (*N* = 106) is described in details in Bastian et al. (submitted, Experiments 1–3). All experiments were based on the Sustained Attention to Response Task (SART), a go/no-go paradigm with rare (<12%) no-go trials. A digit between 0 and 9 was presented for 500 ms every 2000 ms on a computer screen and participants were required to press the space bar as fast and accurately as possible for each digit, but to withhold their response when the number was “3.”

Experiments 1 (*N* = 25) and 2 (*N* = 34) had a within-participants design (respectively 3 conditions—SART single task, SART with articulatory suppression, SART with foot tapping—and 4 conditions—a standard visual SART with reversed speech or white noise and an auditory SART where numbers were displayed through earphones with static or moving random dots on screen). Experiment 3 had a between-participants design: a stereotype threat group (*N* = 15) a no-threat group (*N* = 17), and a public speaking threat group (*N* = 15).

All experiments assessed mind wandering using random thought-probes. Moreover, Experiment 1 and the second part of Experiment 3 also required spontaneous reports of mind wandering as soon as participants were realizing that were mind wandering.

## Contrastive approach

### Data trimming

We focused on (random) reports of on-task thoughts (Nobs = 1302), and on random (Nobs = 902) and spontaneous (Nobs = 564) reports of mind wandering. In two of the three experiments, participants could report that they were experiencing distraction or that they had task-related interferences, but for the present analysis, these reports were discarded.

Our analysis was conducted on the eight trials preceding on-task and off-task thought reports. Thought reports were discarded if these eight trials were not all correct go trials, for example if they contained a no-go trial or an omission (incorrect go trial). Thought reports were also discarded if the eight trials included the first or the second trial of a block, or were interspersed with or immediately preceded by another thought-report (notably when participants where spontaneously reporting mind wandering many times in a row). Indeed the trials immediately following thought reports were significantly slower (640 ms, *SD* = 189) than those immediately preceding thought reports [500 ms, *SD* = 174, *F*_(1, 105)_ = 241, *p* < 0.001] or in second position after them [490 ms, *SD* = 170, *F*_(1, 105)_ = 390, *p* < 0.001], making them unsuitable for an analysis based on response times variability.

A number of 562 on-task thoughts, 384 random reports of mind wandering and 73 spontaneous reports of mind wandering survived data trimming.

### Results and discussion

All data was analyzed using R (R Development Core Team, 2009) with the lme4 (Bates and Maechler, 2009) package for mixed models analyses. All regressions are mixed models with participants, conditions, and experiments as nested random factors, and we present *p*-values that are considered significant at the α = 0.05 level.

#### Contrasting the trials preceding thought reports

First, based on previous evidence that, in the SART, response time variability is higher in trials before no-go errors than in trials before successful withholding of the response (Cheyne et al., 2009), we wanted to assess whether response time variability was similarly higher preceding mind wandering reports compared to on-task reports. We computed the Response Time Coefficient of Variability (RTCV: standard deviation/mean) of the eight trials preceding each probes. RTCV was higher in the eight trials preceding mind wandering reports (0.204, *SD* = 0.108) than in the trials preceding on-task reports (0.171, *SD* = 0.082). We tested this difference in a logistic regression with RTCV as predictor (Nobs = 1019, Nsubj = 106, Ncond = 10, Nexpe = 3). We found that increasing RTCV significantly predicted reports of mind wandering (β = 2.65, *SE* = 0.63, *z* = 4.20, *p* < 0.001). More precise contrasts between on-task reports and random reports of mind wandering (Nobs = 946, Nsubj = 106, Ncond = 10, Nexpe = 3) and between on-task reports and spontaneous reports of mind wandering (Nobs = 635, Nsubj = 103, Ncond = 10, Nexpe = 3) showed that RTCV increased both for randomly probed mind wandering reports and spontaneous reports of mind wandering (respectively β = 2.60, *SE* = 0.68, *z* = 3.82, *p* < 0.001 and β = 3.39, *SE* = 1.07, *z* = 3.16, *p* < 0.01). Moreover, the contrast between random and spontaneous reports of mind wandering (Nobs = 457, Nsubj = 95, Ncond = 10, Nexpe = 3) was not significantly predicted by the RTCV of the eight preceding trials (*p* > 0.9). Table 1 shows that these effects already exist for the four trials preceding the reports and are robust regardless of whether we look at the 5, 6 or 7 trials preceding thought reports.

**Table 1:**
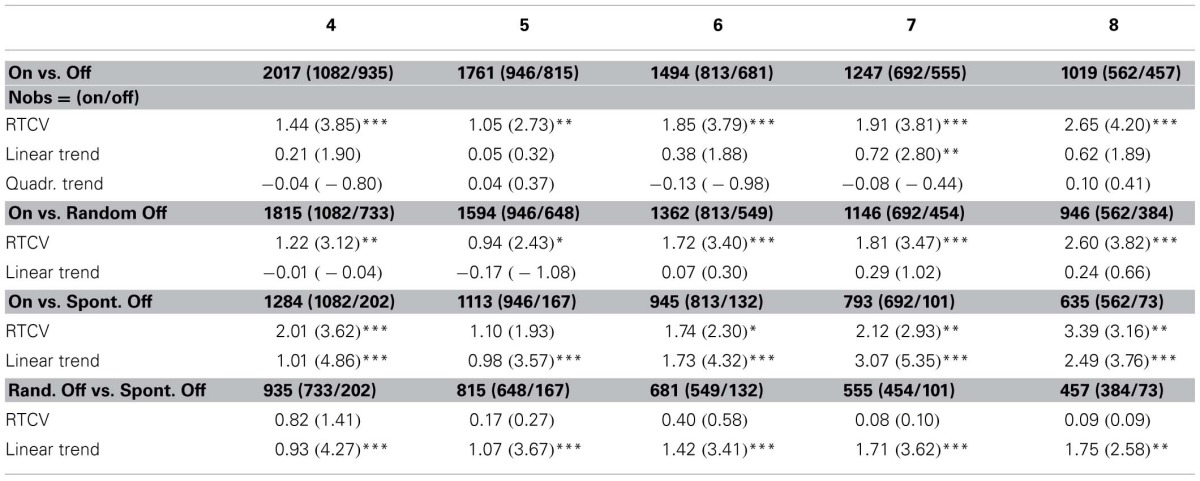
**Contrasting the 4–8 trials preceding mind wandering and on-task reports**.

^*^p < 0.05; ^**^p < 0.01; ^***^p < 0.001.

Second, as the literature has shown specific patterns of response times before errors and mind wandering reports (Smallwood et al., 2008a; Smallwood, 2011; McVay and Kane, 2012), we tested whether the increased variability we found before mind wandering could be accounted for by either linear or quadratic trends. To do so, we normalized response times by condition and by participant using z-scores. Previous studies have used Principal Component Analyses (Smallwood et al., 2008a; Smallwood, 2011; McVay and Kane, 2012), so as identify patterns of response times that were related to mind wandering. Here, we computed the mean of the first differences of the eight trials before a probe [mean of (RT_*n*_ − RT_*n* − 1_) where 8 < *n* < 2] and the mean of the second differences mean of [(RT_*n*_ − RT_*n* − 1_) − (RT_*n* − 1_ − RT_*n* − 2_)] where 8 < *n* < 3), which allowed us to test in a principled way whether linear (first differences) or quadratic trends (second differences) predicted mind wandering reports. In a logistic regression with both the first and second differences as predictors, we found that the second difference was not a significant predictor (*p* > 0.6), but that the first difference marginally predicted mind wandering reports (β = 0.62, *SE* = 0.33, *z* = 1.89, *p* = 0.059), to the effect that participants tended to slow down before a report of mind wandering.

If anything, this tendency goes against the literature that has shown a linear decrease in response times preceding mind wandering episodes (Smallwood et al., 2008a). However, these previous results were obtained with random thought-probes, and not with a conjunction of random and spontaneous reports. To further evaluate this surprising tendency, we separately contrasted random mind wandering reports with on-task reports in a logistic regression with the first difference as predictor, and found no significant effect (*p* > 0.5). As opposed to that, when we separately contrasted on-task reports and *spontaneous* reports of mind wandering, we found that the first difference was highly predictive of mind wandering (β = 2.49, *SE* = 0.66, *z* = 3.76, *p* < 0.001): participants slowed down before a spontaneous report of mind wandering. To test whether this deceleration was specific to imminent spontaneous report, we also contrasted spontaneous with random reports of mind wandering in a logistic regression with the first difference as predictor. We found indeed that a linear deceleration was highly predictive of spontaneous compared to random reports of mind wandering (β = 1.75, *SE* = 0.68, *z* = 2.58, *p* < 0.01). Table 1 shows the robustness of this analysis from the 4 trials preceding thought reports.

Thus, a linear decrease of response times seems specific to impending spontaneous reports of mind wandering. Interestingly, this linear trend is partly dissociated from the general variability of response times as captured by the RTCV. Indeed, in a logistic regression with both RTCV and first difference as predictors, and on-task vs. spontaneous report as outcomes, we found both main effects of linear deceleration (β = 2.22, *SE* = 0.62, *z* = 3.55, *p* < 0.001) and of the RTCV (β = 3.29, *SE* = 1.12, *z* = 2.93, *p* < 0.01). The increase in RTCV preceding spontaneous reports is, therefore not totally captured by the linear deceleration.

To summarize, we first found that high response time variability in the eight trials preceding a thought report was predictive of mind wandering compared to on-task thought. This phenomenon was observed regardless of the method (random or spontaneous reports) used to assess mind wandering. Second, we found that the eight trials preceding spontaneous reports of mind wandering presented a specific pattern of linear deceleration compared to thought reports collected via random thought-probes, regardless of their content (on-task or mind wandering). Hence, although the linear slowing down of response times may be related to consciousness of an episode of mind wandering, it does not appear to be a ubiquitous index of mind wandering. On the contrary, RTCV predicted mind wandering regardless of the method used to assess it. Therefore, RTCV seems a suitable candidate for a continuous index of mind wandering.

#### Contrasting the trials preceding and following thought reports

**Figure 1:**
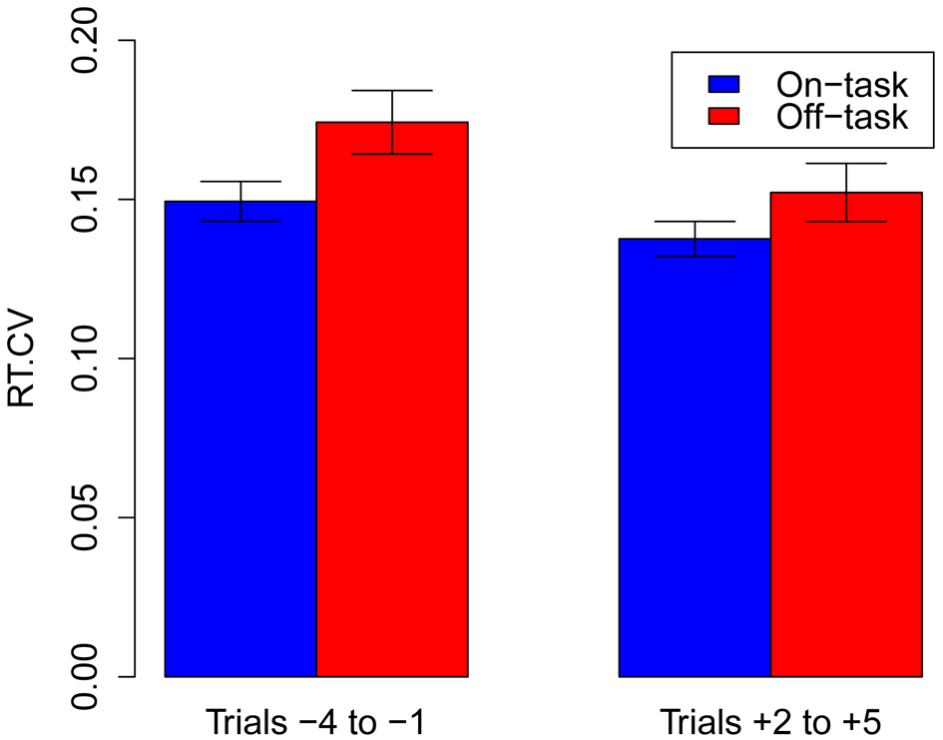
**RTCV (Response time coefficient of variability) as a function of whether participants are on-task or mind wandering (“off-task”), for both the trials preceding and following a report.** Error bars are standard errors of the mean.

Next, we wanted to assess the potential effect of interruptions (spontaneous reports of mind wandering or random external probes) on the RTCV of immediately following trials. As the very first trial after an interruption is significantly slower than the other trials (see Data Trimming), we excluded it and computed the RTCV on the second to fifth trial after an interruption. In order to avoid a null effect exclusively due to excessive data-trimming, we selected reports preceded by four or followed by five correct no-go trials, that were not interspersed with or preceded by an other thought report nor included the first two trials of a block. After trimming, 2508 thought reports were included, 1286 (on-task: 746, off-task: 540) of which had both their 4 preceding trials and their 5 following trials as correct go trials. We then ran a logistic regression contrasting mind wandering and on-task reports, with RTCV and the position of the trials (before vs. after) as predictors. We added the thought report identity as random variable to the other random variables (participant, condition and experiment) since some differences are paired. We found a main effect of RTCV, indicating that higher RTCV was predictive of mind wandering (β = 3.85, *SE* = 1.08, *z* = 3.55, *p* < 0.001), no main effect of position (before/after, *p* > 0.2), and crucially no interaction (*p* > 0.8, see Figure 1). This shows that trials *after* a report of mind wandering are still more variable than trials after an on-task report, which may be indicative that the internal state of participants is not drastically modified by the interruption. Of course we cannot conclude from this null result that participants do not refocus after a report of mind wandering, as we do not have secondary assessment of subjective states after each probe. Perhaps surprisingly, however, this null result indicates that, in our tasks, no-go trials and probes are unobtrusive, so that variability of response times can be used as a *continuous* index of mind wandering.

## Global analysis

### Methods, results and discussion

**Figure 2:**
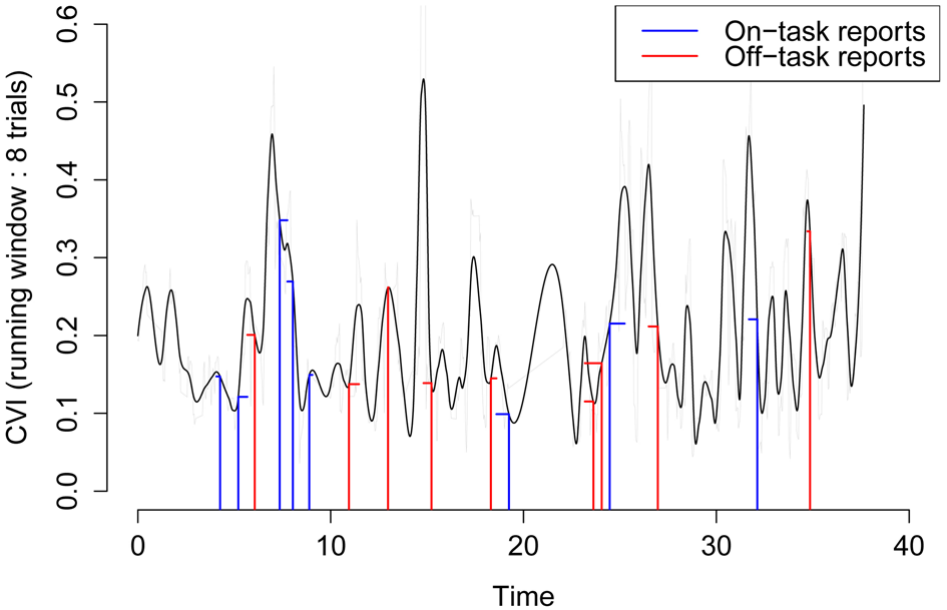
**Distribution of the Continuous Variability Index (CVI) across the experimental session of an arbitrary participant (time in minutes).** Vertical lines represent the CVI value (in RTCV units) at the moment of the report, horizontal lines represent temporal distance from the report to the closest peak in the CVI. We predict that On-task reports (blue) have a lower CVI (shorter vertical lines), and a higher temporal distance to their closest peak (longer horizontal lines) than Off-task reports (red).

Now, we set out to use the RTCV as a continuous index of variability in participants response times. To do so, we removed the first two trials of each experimental block and the first trial after an interruption (random probe or spontaneous report) from the series of correct go responses. On this series, we computed the RTCV within running windows of eight trials, time-stamped to occurrence of the last trial in the window. We then smoothed this index using locally weighted polynomials (LOESS, Cleveland, 1979), to allow interpolations at the moment of reports^1^, yielding a Continuous Variability Index (CVI) that we shall study hereafter. We illustrate the time course of the CVI on Figure 2, for an arbitrary participant.

First, we checked whether the interpolated values of the CVI at the moment of the probes could predict their content. Note that this analysis does not strictly replicate the contrastive analyses presented above, as the dataset here is not trimmed. Notably, running windows on which the computation of the index is done can span across correct no-go trials, incorrect go trials (omissions) and interruptions. We then ran a logistic regression with the content of the report as outcome (on-task: 1302, mind wandering: 1466) with the CVI at the moment of the report as predictor (Nsuj = 106, Nconditions = 10, Nexpe = 3). We found that CVI significantly predicted the content of the report, with higher values being predictive of mind wandering (β = 2.30, *SE* = 0.42, *z* = 5.55, *p* < 0.001).

This result extends the contrastive approach used so far, but does not modify its logic. Now we reasoned that if the CVI does reflect the time-course of subjective states, its critical moments, namely its local extrema (troughs and peaks), might correspond to different probabilities of being in a mind wandering state. More precisely, we predicted that local peaks of CVI might correspond to increased likelihood of an occurrence of a mind wandering episodes. Hence, the temporally closer to peaks, the more likely to report mind wandering. To test this hypothesis, we measured the temporal distance of thought reports to the closest peak in the CVI. We ran a logistic regression with the content of the report as outcome and temporal distance as predictor. We found that temporal distance significantly predicted the content of the report, with lower values (closer to peaks) being predictive of mind wandering (β = −0.43, *SE* = 0.17, *z* = −2.61, *p* < 0.01).

However, on average, by construction, local peaks have higher CVI values than troughs. Thus, the effect of temporal distance just presented may be simply an obfuscated replication of the effect of the CVI *value*. To control for that, we ran a logistic regression on the content of the report with the interaction between CVI at the time of the probe and the temporal distance to the closest peak as predictor. We crucially found that this interaction was negative and highly significant (β = 3.37, *SE* = 0.54, *z* = 6.21, *p* < 0.001). When the temporal distance was null (the probe was on the peak), the CVI was not a significant predictor of mental content (*p* > 0.5), but became so as the probe is farther from the peak. Conversely, when the CVI was null, the temporal distance to the closest peak predicted mental content (β = −1.17, *SE* = 0.29, *z* = −4.03, *p* < 0.001). However, the negative interaction indicates that this temporal effect decreased with increasing CVI. To summarize, both effects were in opposition: the predictive power of temporal distance decreased with increasing CVI, and the predictive power of CVI decreased with increasing closeness to a peak.

Our results build on and extends previous findings: errors are typically preceded by higher response time variability (Cheyne et al., 2009), and higher rate of mind wandering correlate with higher RTCV at the participant level (Hu et al., 2012). Indeed, we show that the prediction of mind wandering through values of RTCV is local (preceding the reports), robust and valid throughout the experiment: we found that *all* the trials of a given SART experiment contain information about mind wandering since the temporal distance to a peaks of variability was indeed a significant predictor of mind wandering. Furthermore, that local fluctuations in variability should be predictive of mind wandering opens the way for on-line detection of mind wandering.

## Hidden markov model of mind wandering fluctuations

### Methods

**Figure 3:**
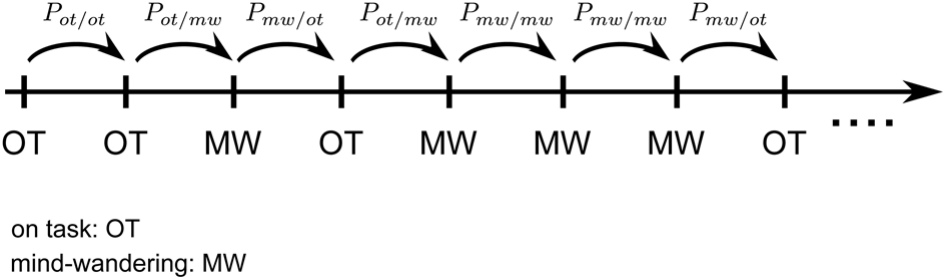
**Markov Chain of attentional states.** Illustrative time series of on-task (OT) and mind wandering (MW) states, with two pairs of complementary probability transitions to stay (ex. *P*_*ot/ot*_: stay focused) in the same state or transition (ex. *P*_*ot/mw*_ = 1 − *P*_*ot/ot*_: start mind wandering) to the other.

The reasoning behind the CVI can be followed-up. We found that mind wandering is characterized by increased response times variability. If we hypothesize that participants are at each time point in one of two distinct states, on-task (OT) or mind wandering (MW), the previous findings suggest that when in each of these states, participants will produce responses according to distinct response generation processes. Based on the previous observations and assumptions, we model the alternation of on-task and mind wandering states. We assume further that OT and MW states have transition probabilities to themselves and the other state, yielding a Markov chain (Figure 3). The notion of a Markov chain is the formal, quantitative counterpart to the intuition that on-task and mind wandering states are organized in runs, so that if at one time point the participant is in one of the two states, it is more probable that he or she should be in the same state at the next time point. The transition probabilities give us a precise estimate of the volatility of each state. Observe two critical points: first, these volatilities are independent from each other, so that for instance OT might be stable (the probability to transition to MW is low), while MW might be more volatile (probability to transition from MW to OT is high). Second, the notion of a Markov chain of attentional states is based on a discretization of time in successive steps. Of course, this is a crude simplification, but it does correspond to the logic of our experiments, which are organized in discrete trials.

**Figure 4:**
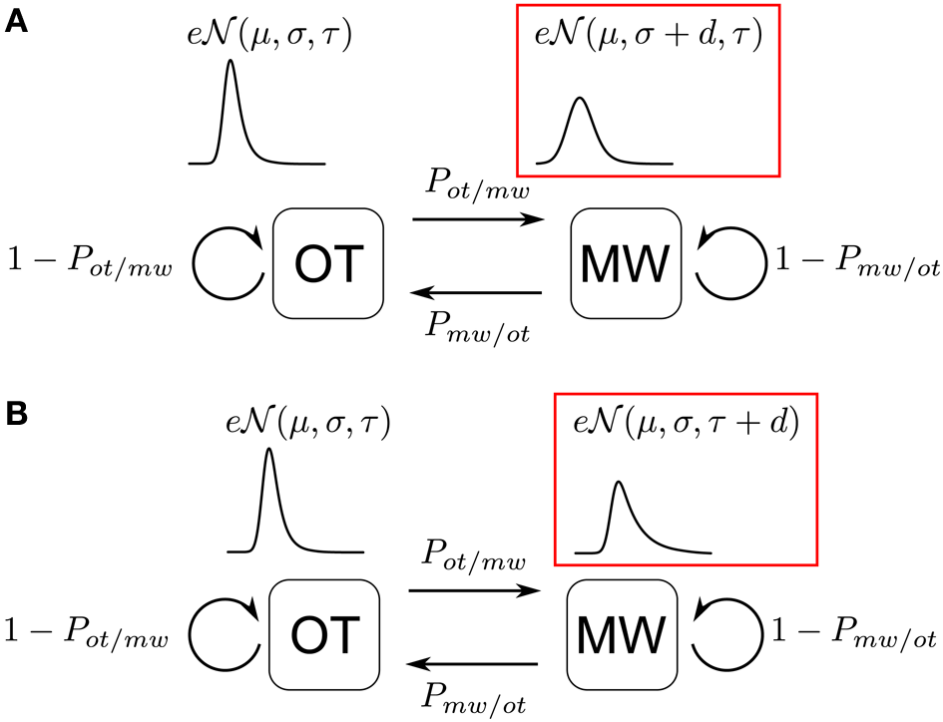
**Six-Parameters Models accounting for increased variability during mind wandering.** *P*_*ot/mw*_: transition probability to start mind wandering when on-task, *P*_*mw/ot*_: transition probability to come back on task when mind wandering, μ: mean of the distribution, σ: variance of the distribution, τ: skewness of the distribution. The critical parameter is *d*, “difference parameter,” applied either to σ (“variance model”) if variance increases during mind wandering, or to τ (“exponential model”) if skewness increases during mind wandering. **(A)** Variance Model **(B)** Exponential Model.

Now, we do not observe directly this Markov chain, but only the response times. However, the preceding sections suggest that in MW, participants generate more variable response times than in OT. Thus, if we can make plausible assumptions about the two distinct response generation processes, we could try to infer the underlying Markov states. This is precisely the logic of Hidden Markov Models (HMMs): to an unobserved Markov chain of two internal states corresponds at each trial an observed output (the response time), which is emitted according to two different probability laws—that here differ according to their variability (see Figures 4A,B).

Thus, one critical step in this model is to characterize the variability of response times. On a descriptive level (Luce, 1986), response times are distributed according to skewed normal laws, meaning that to a bulk of responses that are roughly normally distributed must be added a long “right” tail of slow responses. These properties are nicely captured with the exponentially modified gaussian distribution (ex-gaussian), which is the sum of a gaussian distribution and of an exponential distribution. The parameters of an ex-gaussian are standardly known as the mean (μ) and variance (σ) of the gaussian component, and the rate of its exponential component (τ, which yields the weight of the right hand tail). With this in mind, it is clear that what appears in the CVI as an increase of variability can come from two changes in the ex-gaussian: First, the variance of the gaussian component (σ) could increase, spreading response times around the peak of the distribution. Second, the exponential rate parameter of the distribution (τ) could increase, adding slow response times (see red frames in Figure 4).

Thus, if we are correct in assuming that *variability* is diagnostic of mind wandering, we can conceive of each response time at each trial as a sample from one of two ex-gaussian distributions that differ in their variance or rate parameters. We observe the response times and would like to infer the underlying states that generated them. In a sense, what we are looking for is a partitioning of the trials in latent classes with respect to the distributions of observed response times (see Vandekerckhove et al., 2008 for an example of latent class analysis based on response times). Here, crucially, this partitioning is further constrained by the assumption that the underlying states are organized as a Markov chain.

As a first foray into this kind of analysis of response time series, we tried the simplest and most straightforward models: we assumed that the two emission probability distributions were ex-gaussians with the same mean, and only differed in either the variance of their gaussian component or in the rate of their exponential component. This yielded six free parameters: the transition probabilities of the underlying unobserved Markov chain (*P*_MW → OT_ and *P*_OT → MW_), the three parameters of the base (which we arbitrarily chose as OT) ex-gaussian distribution (μ, σ, τ), and the critical difference parameter *d* that was added either to the variance or the rate of the base distribution, and thus yielded the higher variability MW emission law. If we succeed in so doing, for each trial, the model should yield the posterior probability that the participant should be in OT or MW. In order to account for the fact that trials are not equally distributed in time, and so as to remain within the markovian paradigm, we discretized time in steps of 2 s (equal to the offset of two successive correct go trials). Therefore, most trials would be one Markov transition from each other, but trials farther apart in time (because of correct no go trials, incorrect go trials, or thought probe interruptions) would be separated by a sequence of more than one Markov transitions. We estimated such models for one experiment with 47 participants (Experiment 3 in Bastian et al., submitted).

Our goal was threefold: first, it should yield an independent, principled and confirmatory evidence that variability in response times crucially distinguishes MW from OT states. Second it might help us tease apart the components of variability described and observed in the previous sections: we systematically contrasted a model where the only source of increased variability for the MW state comes from the variance of the gaussian component (hereafter “variance model”) with a model where the increase in variability comes from the rate of the exponential component (“exponential model,” see Figure 4). Finally, and most importantly, we should get an estimate of the volatility of each of the states, through the estimates of the transition probabilities *P*_MW → OT_ and *P*_OT → MW_.

Such models are intractable analytically, but can be implemented as graphical bayesian models (see Lee and Wagenmakers, in press, for an introduction to bayesian graphical models in cognitive science), and can be estimated using Markov Chain Monte-Carlo methods (MCMC). To do so, we used the JAGS software (Plummer, 2003) and the rjags package for *R*. We used uniform priors for all six parameters. We ran two separate models (variance and exponential) for each of the 47 participants, using four MCMC chains of 30000 samples each, with a thinning of 2, and after a burn-in period of 2000 samples.

### Results and discussion

First, we compared the variance and the exponential models using the Deviance Information Criterion (DIC) for each model, for each participant: we computed the difference of DIC for the exponential and variance models, knowing that lower DIC indicates better convergence. The mean DIC difference across participants was −90.2, favoring the exponential model and this difference was significant as shown by a paired Wilcoxon signed rank test (*V* = 247, *p* < 0.001). In other words, the “exponential model” provides a better fit to the data than the “variance model.” Therefore, hereafter we focus on the exponential model^2^.

**Figure 5:**
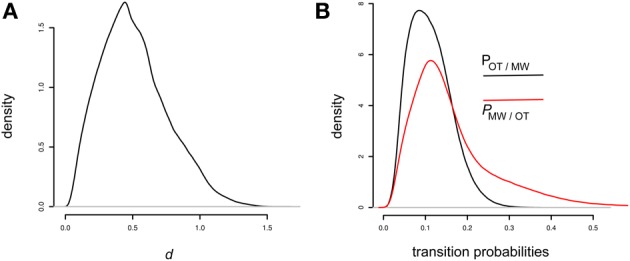
**(A)** Overall posterior distribution of the parameter d in the exponential model, pooled across all 47 participants (60000 samples per participant). Note that the prior was uniform over [0, 2]. **(B)** Overall posterior distribution of the two transition probabilities in the exponential model. The prior was uniform over [0, 1] (60000 samples per participant).

Visual inspection of the sample chains and Gelman diagnostic (Gelman and Rubin, 1992) showed that convergence was attained, therefore it makes sense to interpret the posterior distributions of the parameters. First, as a sanity check, we compared the mean posterior μ and σ with observed participants' mean and variance of response times. The correlations were highly significant [β = 1.2, *t*_(45)_ = 19.44, *p* < 10^−15^, *R*^2^ = 0.89 for the μs/means correlation and β = 0.93, *t*_(45)_ = 4.7 *p* < 10^−4^, *R*^2^ = 0.32 for the σs/variance correlation]. Note that the intercept was significant and positive only for the correlation of σs and variances [41.4, *t*_(45)_ = 4.6, *p* < 10^−4^], which is to be expected because in the model, the exponential parameter adds a further contribution to the observed variance—thus the empirical variance over-estimates the variance component of an ex-gaussian model. These facts suggest that the models did converge on the basic properties of individual response times. Next, we investigated the exponential rate τ and the critical difference parameter *d*. For all participants the model was able to estimate a positive *d*, with a mean of 0.51, and a base τ for the OT state of 0.02. This suggests that the model partitioned the trials in two classes: a class of less variable, quasi-normally distributed response times, and a class of highly variable, heavily skewed response times (see Figure 5A for the overall posterior distribution of *d*).

Now we come to the crucial hidden Markov chain transition probabilities, that we obtained for each participant. The grand mean across all participants of the mean estimates were, respectively 0.11 and 0.18 for *P*_OT → MW_ and *P*_MW → OT_. Critically, this difference was significant as revealed by a paired Wilcoxon test (*V* = 246, *p* < 0.001, see Figure 5B for a plot of the overall posterior distributions of *P*_MW → OT_ and *P*_OT → MW_). Two remarks are in order here. First, these transition probabilities are well below 0.5, meaning that neighboring trials are more likely to belong to the same state than to the opposite state, in agreement with the intuition that mind wandering and on-task states come in stretches longer than our time step of 2 s. Second, and most interestingly, the fact that *P*_OT → MW_ is lower than *P*_MW → OT_ shows that OT is more stable than MW. From this we can estimate the predicted duration of the episodes: in the OT state, if the probability of a transition is 0.11, it means that a transition will occur on average every 1/0.11 = 9.09 steps, that is, every 18.2 s, because of the 2 s time step we used. Similarly, the predicted duration of MW episodes will be (1/0.18) ^*^ 2 = 11.1 s.

**Figure 6:**
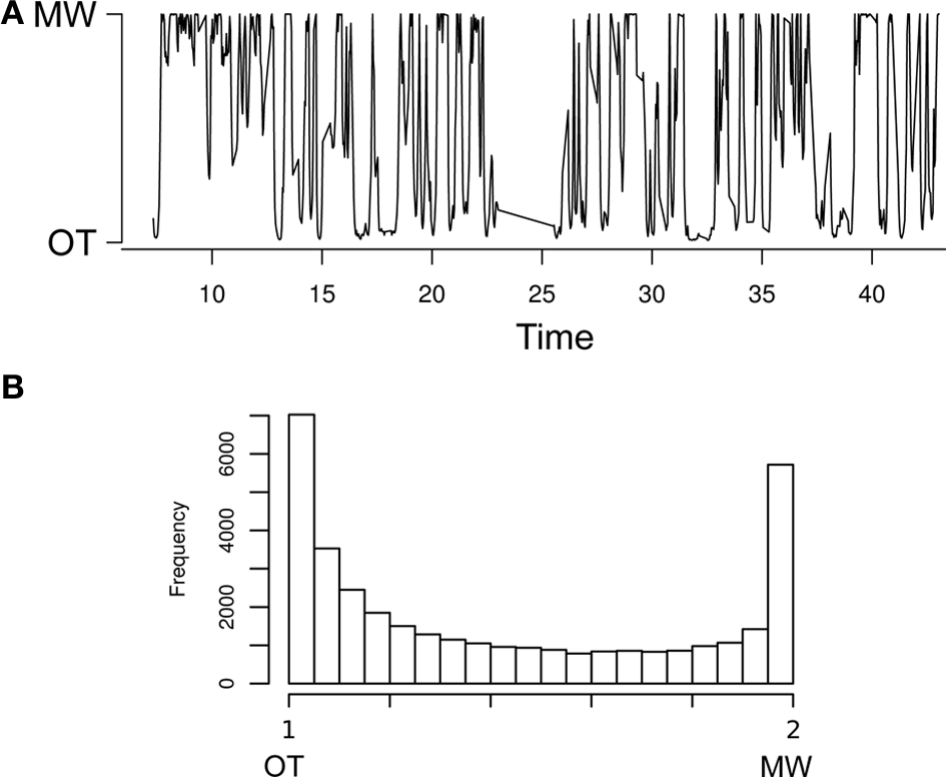
**(A)** Posterior distribution of hidden state across the experimental session of an arbitrary participant (time in minutes). **(B)** Posterior distribution of the hidden states across all participants and trials. This distribution seems bimodal, meaning that the model categorically distinguishes between the two hidden states.

We can now come to the posterior estimates of the underlying Markov states. Recall that for each participant and for each trial, the model computes the posterior probability that the particular response time comes from one or the other (MW or OT) ex-gaussian distribution. We illustrate the time course of these underlying hidden states on Figure 6A, for an arbitrary participant. As is visible on the plot, and as was already clear from the posteriors of *P*_MW → OT_ and *P*_OT → MW_, the model distinguishes runs of OT and MW states. This was confirmed by the fact that the overall (across all participants) distribution of posterior probabilities for the hidden states was bimodal (see Figure 6B): this means that for a clear majority of trials, the model unambiguously assigns each trial to one or the other latent class. It is thus now possible to test whether the states identified by the model correspond to subjective states as experienced by participants. To this end, we applied the same logic as in the previous descriptive sections: first we contrasted the value of the state at the moment of a thought probe when the report is “on-task” to its value when the report is “off-task.” Again, remember that states are only estimated at the moment of correct go trials. Therefore, we needed to interpolate its value at the moment of the probes, which we did by using LOESS smoothing (Cleveland, 1979). Then, we coded OT states as 1 and MW as 2, and computed the median values for each participant separately for mind wandering and on-task reports. This median value was higher (1.44 as opposed to 1.38) when participants reported “off-task,” and this difference was significant according to a two-tailed paired Wilcoxon signed rank test (*V* = 54, *p* < 0.05). This result replicates, in a principled way, the results of the “global analysis” section, where we found that the CVI at the moment of “mind wandering” reports was higher than at the moment of an “on-task” report. In other words, MW states, as identified by the model, correspond to mind wandering in the subjective reports of participants. Thus, not only does the model have internal consistency, in that it succeeds in partitioning trials in two latent classes of differing variability, it also parallels subjective reports of participants.

## General discussion

In this paper, we showed that in a very simple cognitive task, variability of response times is intimately linked to mind wandering. Our paper has both practical and theoretical implications. On the practical side, we contribute to the elaboration and test of a continuous and covert index of mind wandering, that could be used on-line. On the theoretical side, through our model of the fluctuations of variability, we contribute first to the parsing of the components of variability that reflect mind wandering, and second to the distinction of frequency and duration of mind wandering episodes. We will now review the main results we obtained and their implications. We first found that, in the few trials preceding a report, the Response Time Coefficient of Variability (SD/Mean) was highly predictive of the nature of the subsequent thought report. This direct evidence of a relation between RTCV and mind wandering is consistent with previous suggestions on the basis of retrospective reports of mind wandering (Cheyne et al., 2009). Moreover, RTCV was equally predictive of both random and spontaneous reports of mind wandering.

However, we did find a specific pattern of response times before spontaneous reports: our participants systematically slowed down before such reports. Further research is needed to determine the causes of this deceleration. We suggest that it might be due to (a) a dual task cost coming from an upcoming infrequent response; (b) the progressive rise to consciousness of an unconscious mind wandering episode; (c) the start of an episode of conscious mind wandering. Further research is also needed to determine whether this deceleration is the cause of the spontaneous report, as would be the case if for example participants used this information as their decision variable to stop and report their subjective state.

Note that this deceleration before spontaneous reports was the only linear or quadratic trend that we identified as a predictor of mind wandering. McVay and Kane (2012) found that an *acceleration* (cf. also Smallwood et al., 2008a) predicted a subsequent error, but this trend was not predictive of a subsequent report of mind wandering, contrary to what Smallwood and colleagues found (Smallwood et al., 2008a; Smallwood, 2011). The apparent opposition of our results to previous findings is intriguing. Notice, however, that we simply tested the presence of linear or quadratic trends, whereas the descriptive nature of Principal Components Analysis (PCA), the methodology employed notably by Smallwood and colleagues, may help detect more complex patterns. In fact, the PCA factor that is mainly associated with mind wandering (“Factor 2,” in Smallwood et al., 2008a, see their Figure 1) could not easily be described as either simply an “acceleration,” a “deceleration,” or any linear combination of linear and quadratic trends of response times. It seems rather a fluctuation, ending with a deceleration in the three last trials. Thus, it may be that we traded sensitivity for simplicity. As the results obtained by means of PCA and ours are thus not incompatible, further research is clearly needed in order to check whether both generalize to other contexts and tasks.

Next, we found that the CVI, a continuous version of RTCV, was a robust and local predictor of mind wandering: regardless of whether a participant had just gone through a peak of CVI, or was about to reach one, she or he was more likely to report mind wandering as the report (random or spontaneous) was closer to the peak. One may find it surprising that the effect of closeness to a peak of CVI does not depend on whether it is a past peak or whether it is yet to come. It may even seem to run counter to basic principles of metaphysics, as future events are generally not considered as having causal effects back in time (but see Bem, 2011). However, first, in our view, maxima of variability are only points in time when episodes of mind wandering are most likely to occur. Thus, mind wandering could have started before variability in response times reaches its maximum. Second, rhythmic fluctuations have been shown in human vigilance, with periods ranging from 10 s (Fox and Raichle, 2007) to 5 or 30 min (Conte et al., 1995) and even 60–110 min (Okawa et al., 1984). As a consequence, the future of human vigilance seems predictable, and participants need not to be aware of these fluctuations to be anticipating them.

Now, in fact, it is unclear whether participants are in any sense aware of the variability in their response times that we uncovered, and thus whether it has any causal role in their introspection. Perhaps peaks of CVI have a subjective counterpart, hence establishing a graded relation between CVI and mind wandering. High CVI could for example be associated with highly vivid mind wandering, or episodes that would be very likely to reach meta-awareness. However, these are open questions and we do not know whether, in particular, participants use the variability of their response times as a decision variable to spontaneously stop the experiment and report mind wandering. Nevertheless, our results show that continuous tracking of response times variability should be a very simple yet efficient way to detect mind wandering as it unfolds in an experiment. Most importantly, our demonstration that local maxima of response time variability are good indicators of mind wandering shows that the CVI could be used on-line: we do not need to know the grand average of variability in order to decide when it is “high” or “low”: proximity to local maxima is sufficient. Of course, since we showed that future peaks (which would be inaccessible on-line) are also indicative of mind wandering, on-line detection would not be perfect. Yet, our results open the way for the detection of mind wandering in the very first minutes of an experiment, just by tracking the fluctuations in response times variability. We acknowledge that the negative interaction between CVI value and temporal proximity to peaks should be taken into account in an on-line detector of mind wandering: when the absolute variability is “low,” one should rely more on the temporal distance to a peak, but one should neglect the latter when absolute variability is high.

With this in mind, we vindicate a third method for mind wandering studies, in addition to the random probe and the spontaneous reports techniques. Our results show that it is now possible to trigger probes at moments when mind wandering probability is high. This detection technique would share properties with extant techniques: it would be external (as random thought-probes), but it would be unlimited (as spontaneous reports). With detection, one could test hypotheses about mind wandering micro-dynamics with more precision. These dynamics may concern the very occurrence of mind wandering, but also occurrence of its awareness or perception of external stimuli, or perception of time during mind wandering. It would also be critical to study, how mind wandering reacts to systematic detection, with a view to perhaps modify awareness of ones' thoughts or fluctuations in attention. We thus believe that the development of such a method might contribute to the application of mind wandering studies to educational, applied and clinical psychology—as it might help limit the consequence of attentional lapses in industrial settings and offer new avenues for rehabilitation of some attention deficits.

On the theoretical side, we modeled the time series of response times for each participant as a HMM, where the critical variable that distinguishes the hidden states is the variability in the emission law. Of course, intertrial dependencies and sequential effects have been studied for a long time (see for instance Schvaneveldt and Chase, 1969; Gratton et al., 1992), but to our knowledge, our model is the first to extend the logic of intertrial dependence to full time series in psychological experiments (but see Craigmile et al., 2010; see also Killeen, 2013 for the suggestion of applying HMMs *within* trials). We thus moved from the logic where each trial in an experiment is considered as independent, reflecting only the processing triggered by the experimental condition, to a logic where we adopt a *historical* perspective to each experimental run. The model reveals substantive information about response times in general and their relationship with mind wandering.

First, the most surprising discovery is perhaps the fact that the “variance model” provides a far worse fit to the data than the “exponential model.” This suggests that, in our experiments, response time distribution is a mixture of two underlying distributions, a quasi-normal one and a heavily skewed one which, as we seem to see here, corresponds to periods of mind wandering. This nicely fits previous suggestions (McVay and Kane, 2012) that mind wandering is associated with “slow start” trials, during which participants produce abnormally slow responses due to being absent-minded. This also echoes to the finding that ADHD teenagers, who report more mind wandering than control subjects (Shaw and Giambra, 1993), also present more skewed (higher τ) response times distributions than control subjects (Leth-Steensen et al., 2000).

The second important element is more directly related to mind wandering studies. We were indeed able to reproduce in a principled way the association of high variability of response times with subjective reports of mind wandering. This yield a highly interesting perspective on the asymmetry between the two transition probabilities and the associated runs length in the Markov chain of inner states. Remember that we found that OT was more stable than MW, and that as a consequence OT runs were on average longer (18.2 s) than MW runs (11.1 s). This mean duration of mind wandering episodes echoes to previous suggestions based on subjective estimations (Klinger, 1978) and on slow fluctuations in the activation of the default mode network (Fox and Raichle, 2007; Vanhaudenhuyse et al., 2011). However, if our modeling and reasoning are correct, this might the first principled “objective” estimate of the duration and frequency of mind wandering episodes as psychological states. Further researches are needed to test whether specific experimental variables or subjective conditions would separately impact each of the two transition probabilities, leading to various combinations of durations and frequencies for each of the two states.

In conclusion, we acknowledge that all our results are based on the same SART task. Further research is clearly needed to see whether this pattern is specific to this task or whether it generalizes to others. Our entire data set is constituted of *simple* response times, with very limited cognitive processing. The strong relation between response times variability might disappear when more complex cognitive processes are involved during response generation. One may in particular think that, if the variability due to cognitive processing is intrinsically high, it might easily mask differences in variability due to attentional states. In other words, the variability of response times might not be a diagnostic feature of mind wandering with more complex tasks. If this were the case, one should try to determine whether this is due to the fact that, in more complex situations, the component of variability that originates in mind wandering is more difficult to track, or whether it is simply absent.

### Conflict of interest statement

The authors declare that the research was conducted in the absence of any commercial or financial relationships that could be construed as a potential conflict of interest.

## Footnotes

^1^To ensure robustness of the results, we tested a wide range of smoothing parameters. The results presented bellow use a smoothing kernel of 1 min 30 s, but the pattern of results is identical across a wide range of kernels.

^2^Furthermore, none of the properties reported below for the exponential model were found significant with the variance model. Thus the variance model also lacked internal consistency. As a consequence it did not correlate with subjective reports of participants.
